# Striving for Health Equity: The Importance of Social Determinants of Health and Ethical Considerations in Pandemic Preparedness Planning

**DOI:** 10.3389/ijph.2022.1604542

**Published:** 2022-04-05

**Authors:** Hanno Hoven, Nico Dragano, Peter Angerer, Christian Apfelbacher, Insa Backhaus, Barbara Hoffmann, Andrea Icks, Stefan Wilm, Heiner Fangerau, Felicitas Söhner

**Affiliations:** ^1^ Institute of Medical Sociology, Centre for Health and Society, Medical Faculty, Heinrich Heine University Düsseldorf, Düsseldorf, Germany; ^2^ Institute of Occupational and Social Medicine, Centre for Health and Society, Medical Faculty, Heinrich Heine University Düsseldorf, Düsseldorf, Germany; ^3^ Institute of Social Medicine and Health Systems Research, Otto von Guericke University Magdeburg, Magdeburg, Germany; ^4^ Institute for Health Services Research and Health Economics, Centre for Health and Society, Medical Faculty, Heinrich Heine University Düsseldorf, Düsseldorf, Germany; ^5^ Institute of General Practice, Centre for Health and Society, Medical Faculty, Heinrich Heine University Düsseldorf, Düsseldorf, Germany; ^6^ Department of the History, Philosophy, and Ethics of Medicine, Centre for Health and Society, Medical Faculty, Heinrich Heine University Düsseldorf, Düsseldorf, Germany

**Keywords:** health inequalites, social determinansts of health, COVID-19, ethics, pandemic preparedness

## Abstract

Since the WHO’s “Influenza Pandemic Preparedness Plan” in 1999, pandemic preparedness plans at the international and national level have been constantly adapted with the common goal to respond early to outbreaks, identify risks, and outline promising interventions for pandemic containment. Two years into the COVID-19 pandemic, public health experts have started to reflect on the extent to which previous preparations have been helpful as well as on the gaps in pandemic preparedness planning. In the present commentary, we advocate for the inclusion of social and ethical factors in future pandemic planning—factors that have been insufficiently considered so far, although social determinants of infection risk and infectious disease severity contribute to aggravated social inequalities in health.

## Introduction

Since the initial emergence of the novel coronavirus SARS-CoV-2 in spring 2020, a long-term pandemic has developed, posing significant challenges to health systems and affecting people’s daily lives worldwide. To prepare for such situations, pandemic preparedness plans have been developed at various levels in the past. In 1999, for instance, the World Health Organization (WHO) has published the first “Influenza Pandemic Preparedness Plan,” a strategy that has since served as a template for numerous other pandemic preparedness plans at the national level and with the common goal to respond early to outbreaks, identify risks, and outline promising interventions for pandemic containment [1].

To limit risks and harm during a pandemic, pandemic preparedness planning is also continuously adapted, especially after the experience of new local epidemics and pandemics. After the 2009 influenza pandemic, for instance, many countries adapted their pandemic preparedness plans with key changes focusing on intersectoral cooperation, collaboration, disease surveillance and monitoring of countermeasures and strategies for exchanging information and communicating risk [2].

More than 2 years have now passed since the outbreak of the COVID-19 pandemic in 2020, and public health experts have started to reflect on the extent to which previous preparations and pandemic preparedness plans have been helpful, where problems have become apparent, and which aspects have been completely neglected in pandemic management. To this end, various initiatives have been launched to draw early conclusions. The most prominent example is probably the “Independent Panel for Pandemic Preparedness and Response” (https://theindependentpanel.org/) initiated by the WHO. The panel points to the importance of social and ethical issues for pandemic management—both in terms of social inequalities in the risk of infection and morbidity and in terms of social and ethical consequences of non-pharmaceutical pandemic response measures [3]. The goal of the present commentary is to build on the comments of the WHO panel and to explicitly highlight the importance of 1) social determinants of health and 2) ethical considerations for the pandemic response and preparedness from a public health perspective. In doing so, we argue that social and ethical factors remain insufficiently considered in pandemic planning—despite their immense importance for future pandemic management.

## Social Determinants of Health

There is broad consensus that social determinants play a significant role in the development of diseases and are an important contributor to health inequalities. The social gradient in health arises, for instance, from socioeconomic disadvantages, stressful working conditions and low individual health literacy. It has been a long-term goal of the WHO to reduce the social gradient in health and to increase awareness about the social determinants of health. The WHO’s Commission on Social Determinants of Health has, for instance, summarized its calls to reduce the social gradient under the slogan “Closing the gap in a generation” [4]. Various countries have followed and launched strategies to address health inequalities: England, for example, with its “Tackling Health Inequalities” strategy and New Zealand with “Achieving Equity in Health Outcomes”. However, despite this increasing awareness, sustainable success has not yet been achieved and health continues to be unequally distributed worldwide [5].

With respect to communicable diseases (i.e., infectious diseases), specifically, findings from previous pandemics suggest that social determinants of infection risk and disease severity contribute to aggravated social inequalities in health and thus widen the health gap (e.g., [6]). However, these findings are rarely acknowledged, neither in the public discussion nor in the planning and implementation of infection control measures. Especially at the beginning of the SARS-CoV-2 pandemic it was a common narrative to speak of an “equalizing pandemic” in which “everyone was affected equally.” Despite the evidence on their existence, social inequalities in infectious disease came only gradually into public awareness. Only after a few months, first evidence of an increased COVID-19 incidence in poor neighbourhoods and in black communities became available from the United States. As the pandemic unfolded, a series of further analyses from different countries investigating social determinants (e.g., income, education, occupation, and ethnicity) of infection risk and disease severity confirmed this picture. Researchers now increasingly put forward that both the infection risk and the risk of a severe disease course are unequally distributed, with socially disadvantaged groups being at exceptionally high risk (e.g., [7]). Consequently, there is a need to consider vulnerable groups in pandemic preparedness planning. Important social determinants of the spread of infection and individual infection risks are, for example, crowded living conditions and low quality housing, disadvantaged socioeconomic position, low income and low education. Occupation is another domain of potentially elevated risk of exposure. High-risk occupations in a pandemic are those that cannot easily be carried out from home and that are marked by close contacts with other people. Examples include health care workers, first responders or retail workers. Similarly, with regard to disease severity, social disadvantages have an impact on susceptibility and COVID-19 progression [8]. Importantly, they are also strongly associated with higher prevalence of non-communicable disease, which is itself a risk factor for susceptibility and a more severe disease progression. Pre-existing medical conditions are more frequent among socially deprived groups leading to a higher likelihood of severe disease progression and we have, hence, to consider a “double burden of disease.” Lower health literacy as well as deprived health care access and utilization further aggravate inequalities in health outcomes after SARS-CoV-2 infection. However, research on the mechanisms explaining the social gradient in infection and disease needs to be intensified urgently.

Yet, national and international pandemic plans almost completely disregard social determinants as drivers of disease occurrence and progression—clearly at stake with WHO health goals and criticized by researchers for years [9–11]. The practical consequences are far-reaching with many countries lacking a social-epidemiological surveillance system that helps to early identify population groups at greatest risk of infection. Consequently, data on social determinants of health is lacking, especially when it comes to integrated reporting of social and health data in municipalities to identify disadvantaged population groups at early stages of a pandemic.

Taking social determinants into account already in pandemic preparedness plans may lead to better planning, not least in the provision of intensive care capacities or vaccination strategies but also in risk communication [12]. Infection control measures need to be sensitive to social differences in the exposure to the virus, and social distancing measures need to consider the limiting effects of work environments, including job security and income replacement. Free access to health care, finally, needs to be ensured, especially in deprived neighbourhoods. This surely includes equal access to diagnostic tests, vaccination campaigns, and primary care.

Importantly, from a Public Health perspective, data on the social determinants of infection and disease progression should be collected in a structured manner by established national and international data platforms. For these platforms, it is imperative to include data on social determinants of health to identify detailed social patterns in infection risk and disease progression. To achieve this aim, long-term clinical data collection initiatives and population-representative samples are needed to estimate the infection risk among different social groups. Social situation-sensitive interventions (e.g., protective measures for high-risk occupations, specific lockdown measures, and prioritization of vulnerable groups within vaccination campaigns) rely on knowledge about the population groups being at highest risk.

## Ethical Considerations

Ethical values and principles are particularly at stake when a pandemic response requires drastic pandemic prevention or intervention measures, including, for example, restrictions in the freedom of assembly, in opportunities for social interaction, and economic activity. Currently implemented non-pharmaceutical interventions (e.g., economic lockdowns, social distancing, travel restrictions) are intended to prevent regionally occurring epidemics from developing into pandemics. While on the one hand, this can help to contain the spread of infectious diseases, there are enormous social and economic consequences on the other hand. Here, questions of the required infection control measures are opposed to the social need for a minimum of interpersonal contact. Hence, policy-makers are confronted with fundamental ethical issues at national and international level, including: 1) equal, equitable, and cost-effective access to limited health resources in the event of increased demand and possible bottlenecks, 2) the obligations of medical personnel in the face of risks to their own health, 3) the balance between reducing the spread of disease through isolation measures and protecting individuals’ rights of free movement, and 4) in times of limited resources ethically balanced criteria for resource allocations [13].

Although basic ethical issues are considered in existing pandemic preparedness plans, they still lack a comprehensive framework and a sufficient discussion of ethical dilemmas often arising during pandemics. Such an ethical framework is particularly difficult to maintain since pandemic management constantly requires rapid decision-making. Conflicts between safeguarding the functioning of the health care system and the side effects of far-reaching infection control measures as well as the relative importance of different moral goods need, however, to be negotiated [14].

In this context, the need to discussing existing pandemic plans and necessary improvements to clarify ethical issues were emphasised by the WHO (2). Using the pandemic influenza preparedness planning as a guide, the WHO Eleventh Futures Forum 2008 discussed general and overarching ethical leadership principles for health systems in the European Region. The aim was to enable stakeholders to identify good practices, share experiences on ethical leadership in preparedness planning and identify existing knowledge gaps. This involved examining the ethical considerations in preparedness planning in the European Region and, specifically, ethical frameworks from Norway, Switzerland and the United Kingdom [13]. In Switzerland, for example, the Federal Office of Public Health worked on a national preparedness plan in 2005, discussing a set of values—such as solidarity, individual freedom, proportionality, privacy, fairness and trust. In Norway, the Institute of Public Health and the Directorate of Health and Welfare emphasized on issues of prioritisation in pandemic influenza planning and formulated population categories of different priority. A step forward is also the United Kingdom’s special committee on ethical aspects of pandemic influenza (CEAPI), agreeing on the following ethical principles to be included into pandemic preparedness planning: 1) Treating people with concern and respect, 2) Minimizing harm, 3) Fairness, 4) Working together, 5) Reciprocity, 6) Keeping things in proportion, 7) Flexibility, and 8) Good decision-making [13].

Similarly, based on the experiences of the Ebola outbreak 2005 in West Africa, Smith and Upshur critically reflected on repeated moral failures like inequitable health priorities or inequitable institutional structures [15]. They formulated a binding set of values that embodies a sense of solidarity and global justice, highlighted the need for ethical aspects to be integrated in pandemic preparedness planning and addressed the uncertainty of research, the duty of care and protection in pandemic management, equal distribution of resources and global governance based on solidarity [15]. Importantly, ethical aspects should consider the successive phases of pandemics (interpandemic and alert phase, pandemic phase, transition and interpandemic phase), each involving different ethical issues (e.g., vulnerability and systemic relevance, social distancing measures, quarantine, vaccination measures and distributive justice) [16].

However, although ethics committees, advisory groups or ethics commissions at national level have been established worldwide and although plenty of research has been done on ethical guidelines, ethical dimensions have not been explicitly integrated into pandemic plans. Ethical frameworks such as the above described CEAPI principals that have been established for pandemic influenza preparedness need to be transferred to a more general framework of pandemic response and future pandemic planning. An example of how this can be done is the Nuffield Council’s report “Research in Global Health Emergencies” with the aim to identify ways in which research in emergencies can be conducted from an ethical perspective. The report presents these values in the form of an “ethical compass” to guide the behaviour of the many people involved in research in worldwide health emergencies [17]. To summarize, the recognition of the need for ethical decision-making is highly important: Public discussion and early consideration of ethical issues in pandemic management can help to maintain public trust and promote compliance [15]. Ethical debates can help to identify the goods and values affected and weigh the rights and interests involved at the individual, institutional, and societal level [18]. However, policy-makers need to be aware that some of the internationally accepted principles may still need to be balanced at the national level (e.g., solidarity and respect for individual choices). Successful pandemic management, therefore, addresses ethical implications early and deliberately in the preparation of pandemic plans and provides ethical consideration already in the development of intervention catalogues.

## Conclusion

**FIGURE 1:**
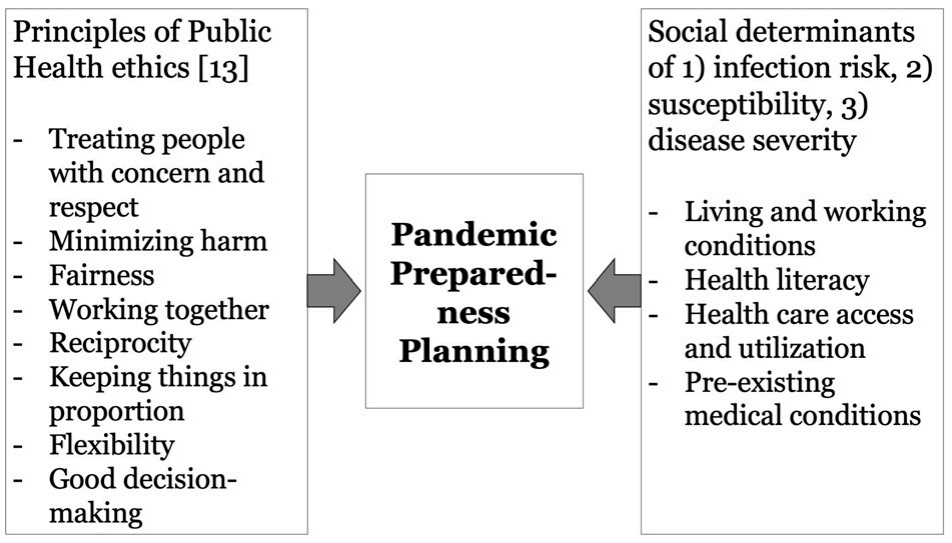
Ethical and social considerations in pandemic preparedness planning (Düsseldorf, Germany. 2022).

Addressing social determinants of health and ethical considerations in pandemic preparedness plans contributes to successful pandemic management at multiple levels (Figure 1). In fact, identifying vulnerable groups and populations at risk is crucial for reducing the social gradient in health during a pandemic. Specifically, identifying risk groups along social categories helps to address chains of infection and allows health care capacity planning. Addressing ethical issues may help to increase the acceptance of infection control measures. In conclusion, social and ethical issues need to be integrated into pandemic preparedness plans so that they are recognized and addressed in all policies, 1) by including them in regular independent parliamentary reports, 2) *via* better preparation and training of health workers and decision makers, and 3) by integrating findings from interdisciplinary implementation science, as well as health communication experts.

## Data Availability

The original contributions presented in the study are included in the article/Supplementary Material, further inquiries can be directed to the corresponding author.
